# Combined Process of Biogenic Manganese Oxide and Manganese-Oxidizing Microalgae for Improved Diclofenac Removal Performance: Two Different Kinds of Synergistic Effects

**DOI:** 10.3390/toxics10050230

**Published:** 2022-04-30

**Authors:** Quanfeng Wang, Cenhui Liao, Jujiao Zhao, Guoming Zeng, Wenbo Liu, Pei Gao, Da Sun, Juan Du

**Affiliations:** 1 School of Civil Engineering and Architecture, Chongqing University of Science and Technology, Chongqing 401331, China; 18046504243@163.com (Q.W.); 2021206106@cqust.edu.cn (C.L.); zeng373064894@126.com (G.Z.); rococo_gao@163.com (P.G.); 2 College of Environment and Ecology, Chongqing University, Chongqing 400045, China; wenbo.liu@cqu.edu.cn; 3 Institute of Life Sciences & Biomedical Collaborative Innovation Center of Zhejiang Province, Wenzhou University, Wenzhou 325035, China; 4 School of Pharmacy and Nusing, Chongqing Vocational College of Light Industry, Chongqing 400065, China; 18360860982@163.com

**Keywords:** synergistic mechanisms, manganese-oxidizing microalgae, biogenic manganese oxides, diclofenac, degradation pathway

## Abstract

Biogenic manganese oxides (Bio-MnOx) have attracted considerable attention for removing pharmaceutical contaminants (PhCs) due to their high oxidation capacity and environmental friendliness. Mn-oxidizing microalgae (MnOMs) generate Bio-MnOx with low energy and organic nutrients input and degrade PhCs. The combined process of MnOMs and Bio-MnOx exhibits good prospects for PhCs removal. However, the synergistic effects of MnOMs and Bio-MnOx in PhCs removal are still unclear. The performance of MnOMs/Bio-MnOx towards diclofenac (DCF) removal was evaluated, and the mechanism was revealed. Our results showed that the Bio-MnOx produced by MnOMs were amorphous nanoparticles, and these MnOMs have a good Mn^2+^ tolerance and oxidation efficiency (80–90%) when the Mn^2+^ concentration is below 1.00 mmol/L. MnOMs/Bio-MnOx significantly promotes DCF (1 mg/L) removal rate between 0.167 ± 0.008 mg/L·d (by MnOMs alone) and 0.125 ± 0.024 mg/L·d (by Bio-MnOx alone) to 0.250 ± 0.016 mg/L·d. The superior performance of MnOMs/Bio-MnOx could be attributed to the continuous Bio-MnOx regeneration and the sharing of DCF degradation intermediates between Bio-MnOx and MnOMs. Additionally, the pathways of DCF degradation by Bio-MnOx and MnOMs were proposed. This work could shed light on the synergistic effects of MnOMs and Bio-MnOx in PhCs removal and guide the development of MnOMs/Bio-MnOx processes for removing DCF or other PhCs from wastewater.

## 1. Introduction

The occurrence of pharmaceuticals and personal care products (PPCPs) in the aquatic environment has attracted increasing concern due to their environmental fate and toxicological properties [1]. Within PPCPs, the pharmaceutical contaminants (PhCs) represent an especially worrying class. Although the environmental concentration of most PPCPs is very low (ng/L to μg/L range), they are designed to cause a physiological response; thus, they can also cause various adverse effects on non-target individuals and species, including humans [2]. Among PPCPs, diclofenac (DCF) is a non-steroidal anti-inflammatory drug among the most consumed drugs in the world. Owing to its wide use and resistance to biodegradation, DCF is frequently detected in various aquatic environments [3]. In addition, it has been reported that the accumulation of DCF in kidneys may be associated with kidney failure in fish and vultures of the genus Gyps [1]. Therefore, alternative strategies must be conducted for PhCs (e.g., DCF) removal from wastewaters.

Manganese oxides (MnOxs) have been widely used as excellent adsorbents, oxidants, or catalysts in the removal of PhCs [4,5]. Among them, biogenic manganese oxides (Bio-MnOx) produced by microorganisms (including bacteria, fungi, and microalgae) generally present a stronger oxidation capacity and higher specific surface area than the abiotic MnOxs, leading to the unique advantages of Bio-MnOx for pollutants removal [6,7]. Moreover, Bio-MnOx production and PhCs removal by Bio-MnOx can be conducted under environmentally mild conditions (e.g., neutral pH and ordinary temperature) [8]. Therefore, as an environmentally-friendly and cost-effective biomaterial that can remove PhCs, Bio-MnOx has attracted increased interest over the past few decades [8].

According to previous studies, Bio-MnOx could effectively remove a variety of PhCs, including ciprofloxacin [9], paracetamol [10], sulfamethoxazole [11], 17α-ethinylestradiol [12], ofloxacin [13], diclofenac (DCF) [14], and other antibacterial agents [4]. Since microorganisms generate Bio-MnOx, many researchers have focused on the removal of PhCs by the combined process of Bio-MnOx and microorganisms [9]. For instance, manganese-oxidizing bacteria (MnOBs) and Bio-MnOx may form a combined process for PhCs removal [12,14]. MnOBs can continuously re-oxidize the Mn(II), which is generated from the redox reaction between PhCs and Bio-MnOx, producing new Mn(III, IV) oxides for additional pollutants removal [4]. Due to this synergistic effect, MnOBs/Bio-MnOx process showed better performance than either MnOBs or Bio-MnOx for PhCs removal [12]. However, the performance of MnOBs/Bio-MnOx for PhCs removal is still not satisfactory, and further improvement is needed.

One important limitation of MnOBs/Bio-MnOx is that most MnOBs cannot degrade PhCs by themselves. Treated by MnOBs/Bio-MnOx, PhCs are mainly removed through the abiotic oxidation capacity of Bio-MnOx, whereas MnOBs only participate in the continuous regeneration of Bio-MnOx [15]. This demerit significantly hinders the performance improvement of the MnOBs/Bio-MnOx process. Besides, aeration and supplementary organic nutrients are necessary for producing Bio-MnOx by Mn-oxidizing bacteria/fungi, which increases their operation cost and limits PhCs removal applications. Therefore, the combined process of microorganisms that can degrade PhCs and low energy input is highly demanded.

PhCs removal by microalgae has recently gained increasing attention because this process can simultaneously remove PhCs and produce bio-energy and valuable products [16]. It has been reported that different microalgae can effectively remove a variety of PhCs, such as carbamazepine [17], sulfamethazine [18], and DCF [2]. In addition, several recent studies have reported some manganese-oxidizing microalgae (MnOMs), which can oxidize Mn^2+^ to Bio-MnOx through direct (generation of specific oxidizing enzymes) or indirect (e.g., generation of oxygen and alkaline) mechanisms [19,20]. Thus, with the functional microalgae that can remove PhCs and produce Bio-MnOx simultaneously, the novel MnOMs/Bio-MnOx process removes PhCs through biodegradation and chemical oxidation, implying superior performance in PhCs removal [6,21]. Moreover, the production of Bio-MnOx by microalgae does not require aeration or supplementary organic nutrients, which favors practical applications of the MnOMs/Bio-MnOx process. However, despite the unique advantages exhibited by MnOMs/Bio-MnOx, mechanisms related to the mutual influence between Bio-MnOx and MnOMs and the pathways of PhCs degradation are still unclear, limiting further development of the MnOMs/Bio-MnOx process.

The effect of Mn^2+^ concentration on the growth of MnOMs is one of the key factors that significantly influence the stability of the MnOMs/Bio-MnOx process. Mn^2+^ is the source necessary for producing Bio-MnOx; however, it may hinder the growth of MnOMs due to its biotoxicity. More importantly, the synergistic effects of MnOMs and the in situ generated Bio-MnOx for PhCs removal are also unclear. In the MnOMs/Bio-MnOx process, biodegradation and chemical oxidation happen simultaneously, so the intermediates generated from the oxidation of PhCs might be continuously degraded by Bio-MnOx and MnOMs alternatively. This potential process may result in different degradation pathways of PhCs and facilitate the removal of PhCs. However, few studies have mentioned these mechanisms.

DCF, a typical PhC frequently detected in different aquatic environments due to its being refractory to conventional biological processes, was chosen as the target pollutant [22]. A mixture of MnOMs (including *Chlamydomonas* sp. WH1-1, *Chlamydomonas* sp. WH1-4, *Chlorella* sp. WH2-4, and *Chlorella* sp. WH2-5) with the ability to degrade DCF was selected to form the MnOMs/Bio-MnOx process. Our aims for the present study were to: (i) Assess the effects of Mn^2+^ on the growth, total chlorophyll content, photosynthetic activity, and Mn^2+^-oxidizing capacity of mixed MnOMs; (ii) evaluate the DCF removal efficiency and investigate the degradation pathway of DCF and the synergistic mechanisms of Bio-MnOx and MnOMs accelerating DCF removal.

## 2. Materials and Methods

### 2.1. Manganese-Oxidizing Microalgae and Media

Four strains of MnOMs were isolated from two aquatic environments in our previous study, namely *Chlamydomonas* sp. WH1-1, *Chlamydomonas* sp. WH1-4, *Chlorella* sp. WH2-4, and *Chlorella* sp. WH2-5 [6]. In experiments for assessing the effects of Mn^2+^ on microalgal growth, total chlorophyll content, photosynthetic activity, and Mn^2+^-oxidizing capacity of mixed MnOMs, and for investigating the DCF removal by MnOMs and/or Bio-MnOx, the BG-11 media were used as described in our previous study. The details of the recipe can be found in Text S1. The stock solution of DCF or MnCl_2_ was added into the BG-11 medium with the final desired concentrations.

### 2.2. Experimental Setup

#### 2.2.1. Mn^2+^ Tolerance and Oxidation Ability of MnOMs

The same amount of four strains of MnOMs was first mixed and then incubated to evaluate the influence of Mn^2+^ on the growth, total chlorophyll content, photosynthetic activity, as well as Mn^2+^-oxidizing capacity of the mixed MnOMs. The mixed strains were then added into a sterilized BG-11 medium. The mixed MnOMs suspension was used as an inoculum in the following experiments by adjusting its optical density to 1.0 at 680 nm (namely OD_680_ = 1.0) using a sterilized BG-11 medium. The mixed MnOMs inoculum (OD_680_ = 1.0, 5% *w*/*w*) were added into a 250 mL Erlenmeyer flask with a total of 100 mL solution. The Mn^2+^ concentrations were adjusted from 0.25 mmol/L to 2.00 mmol/L by adding a certain amount of MnCl_2_ stock solution (0.1 M) into the system. The mixed MnOMs were incubated under artificial sunlight with an intensity of 60 μmol/m^2^/s. The temperature was maintained at 27 °C. The light/dark ratio during the experiment was 12:12 h; the flasks were shaken manually every 6 h to distribute MOMs in the suspension uniformly.

During the 10-day incubation experiment, the samples of microalgal suspension were gathered every two days to measure the microalgal growth, total chlorophyll content, photosynthetic activity of MnOMs, Bio-MnOx concentrations, and Mn^2+^ concentrations. The samples for analysis of DCF metabolites were obtained at the end of incubation and finally analyzed by a high-performance liquid chromatography-tandem mass spectrometry (HPLC–MS/MS).

Along with the experiments, negative controls, Mn^2+^-free or algae-free, were also performed under the same conditions. All experiments were conducted in triplicate.

#### 2.2.2. Removal of DCF by MnOMs and/or Bio-MnOx

Batch experiments were employed to evaluate the removal of DCF by MnOMs, Bio-MnOx, and MnOMs/Bio-MnOx. In the experiment referring to the DCF removal by MnOMs, a certain volume of DCF stock solution was added to the BG-11 solution containing 5% of the mixed microalgal suspension (OD_680_ = 1.0) to obtain the DCF concentration of 1 mg/L. The entire volume of the solution was 100 mL and stored in a 250-mL Erlenmeyer flask. To evaluate the DCF removal by Bio-MnOx, MnOMs were incubated in 100 mL of BG-11 medium with 1.00 mmol/L of Mn^2+^ for 10 days. Afterward, the Bio-MnOx was collected by removing the MnOMs from the modified cell lysis method [17] (details in Text S2). The produced Bio-MnOx was collected via centrifugation and freeze-dried. The obtained Bio-MnOx was then dropped into the BG-11 medium with 1 mg/L of DCF; the initial concentration of Bio-MnOx was about 0.9 mM. The investigation into the DCF removal by MnOMs/Bio-MnOx was conducted in 100 ml BG-11 medium containing 1.00 mmol/L of Mn^2+^, 5% of mixed microalgal suspension (OD_680_ = 1.0), and 1 mg/L of DCF. In this experiment, the gradual generation of Bio-MnOx, and the growth of MnOMs, were recorded. The concentration of MnOMs will reach and maintain a certain value at the end of the experiments. All experiments were performed under similar conditions for 10 days (temperature: 27 °C; the intensity of light: 60 μmol/m^2^/s; the light:dark ratio: 12 h/12 h). Blank samples with no MnOMs or Bio-MnOx were also prepared. The aliquots were samples obtained on 0, 2, 4, 6, 8, and 10 days to analyze the concentrations of DCF, Mn^2+^ in the solution, and Bio-MnOx. 

### 2.3. Analysis Methods

#### 2.3.1. Measurements of MnOMs Growth, Total Chlorophyll, and Photosynthetic Activity

The OD_680_ of the microalgal suspension was used to evaluate microalgal growth [17], and DR2000 (HACH, Loveland, CO, USA) recorded the value of OD_680_. For batch experiments with Mn^2+^, the microalgal suspension was sampled and analyzed for OD_680_ after adding ascorbic acid (0.1 mol/L) to exclude the Bio-MnOx. The total chlorophyll and carotenoid contents in the microalgae were measured as described previously [17]. The photosynthetic activity was revealed by the OJIP-transient (OJIP test). The detailed description for measuring total chlorophyll and photosynthetic activity can be found in Text S3.

#### 2.3.2. Measurement of Mn^2+^ and Bio-MnOx

The inductively coupled plasma (ICP) spectrometry was used to quantify the dissolved residual Mn^2+^, whereas the LBB spectrophotometry was used to quantify the generated Bio-MnOx [6]. The total removal efficiency of Mn^2+^ can be calculated (Equation (1)), as well as the total Mn^2+^ oxidation efficiency (Equation (2)), using the amount of removed Mn^2+^ and generated Bio-MnOx.

(1) $$R_{t}=\frac{\left(C_{i}-C_{r}\right)}{C_{i}}\times 100\%$$

(2) $$R_{o}=\frac{C_{Bio-MnOx}}{C_{i}}\times 100\%$$

where *R_t_* is the total Mn^2+^ removal efficiency. *C_i_* and *C_r_* are the initial Mn^2+^ concentration and the residual Mn^2+^ in the medium, respectively. Ro is the total Mn^2+^ oxidation efficiency and *C_Bio-MnOx_* is the concentration of generated Bio-MnOx.

#### 2.3.3. Characterizations of Bio-MnOx

We used field-emission scanning electron microscopy (FE–SEM) to characterize the Bio-MnOx produced by MnOMs with a SU8020 FE-SEM (HITACHI, Tokyo, Japan). The X-ray diffraction (XRD) pattern of Bio-MnOx was recorded by a BRUCKER D8 ADVANCE diffractometer (BRUCKER, Germany). A detailed description of the measurement methods was reported in our previous study [6]. The FE–SEM and XRD results indicated that the Bio-MnOx produced by MnOMs were wrapped around the microalgal and were nano-sized, poorly crystallized, or amorphous particles (Figures S1 and S2).

#### 2.3.4. Measurement of the DCF Concentration and Intermediates Identification

The DCF concentration was analyzed by an HPLC system (Agilent 1260, Agilent Technologies, Santa Clara, CA, USA) equipped with a Zorbax Eclipse XDB-C18 column (2.1 × 100 mm, 3.5 μm, Agilent). The HPLC–MS/MS (Agilent 1290/6420MSD) was employed to detect the DCF intermediates. Mass spectrometry was recorded using positive electrospray ionization with a mass scan range of *m*/*z* 50–1000. The HPLC and HPLC-MS/MS parameters for DCF and its metabolite analysis were referenced from the previous study [23] (our detailed methodology can be found in Text S4). The DCF adsorbed and bioaccumulated by microalgal cells was also quantified according to the method in our previous paper [14] (details are in Text S5). By adding ascorbic acid (0.1 mol/L) to dissolve the Bio-MnOx, the DCF adsorbed by Bio-MnOx releases into the liquid phase and can be analyzed.

### 2.4. Statistical Analysis

The significant differences in the MnOMs growth, Mn^2+^ oxidation efficiency, chlorophyll content, photosynthetic activity, and DCF removal efficiency were assessed using a one-way analysis of variance (ANOVA) and the Fisher’s least significant difference (LSD) post hoc (α = 0.05) test. These calculations were conducted in Origin 9.0 software.

## 3. Results and Discussion

### 3.1. Mn^2+^ Tolerance and Mn^2+^ Oxidation Ability of MnOMs

#### 3.1.1. Effect of Mn^2+^ on MnOMs Growth and Chlorophyll Content

**Figure 1:**
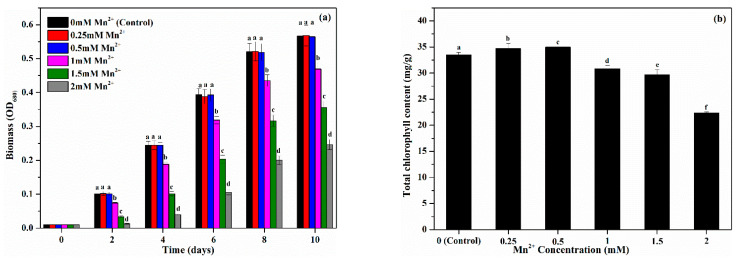
Effect of Mn^2+^ on (**a**) the mixed MnOMs growth, and (**b**) the total chlorophyll content after 10 days of cultivation. The different letters above the error bars showed significant differences between control and treatment (*p* < 0.05).

It is widely speculated that the mixture of different microalgae strains could present better resistance to the biotoxicity induced by metal ions [19]. In this study, the effect of Mn^2+^ on the mixed MnOMs was evaluated for the first time. As shown in Figure 1, the growth of mixed MnOMs had no significant difference (*p* > 0.05) at a low concentration range of Mn^2+^ (0–0.50 mmol/L), whereas increasing the Mn^2+^ concentration (>0.50 mmol/L) would significantly (*p* > 0.05) inhibit the MnOMs growth. This result was similar to our previous study concerning the respective effects of Mn^2+^ on these four microalgae [6], indicating that mixing the MnOMs cannot improve their tolerance to Mn^2+^. As an essential cofactor in the photosystem II (PSII), manganese plays an important role in the photosynthesis of microalgae [24]. However, overly high Mn concentrations can inhibit the microalgal metabolism and biochemical composition biosynthesis (e.g., chlorophyll) [25]. A high concentration of Mn^2+^ (>0.50 mmol/L) decreases the chlorophyll content of the MnOMs (Figure 1b). Chlorophyll is an important photosynthetic pigment and a protective agent that scavenges the over-generated ROS in microalgal cells due to the stress of xenobiotic compounds, such as heavy metals and PhCs [26,27]. Therefore, reduced chlorophylls may be a reason for inhibiting MnOMs growth at high Mn^2+^ concentrations.

#### 3.1.2. Effect of Mn^2+^ on the Photosynthetic Activity of MnOMs

**Figure 2:**
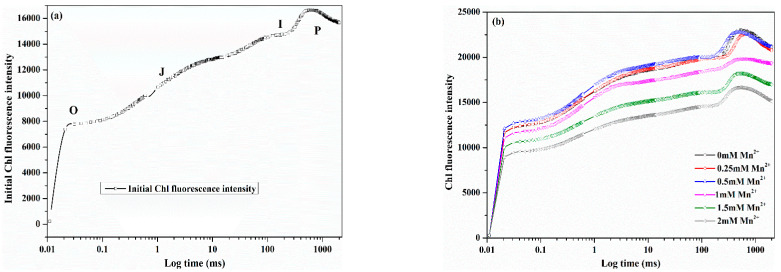

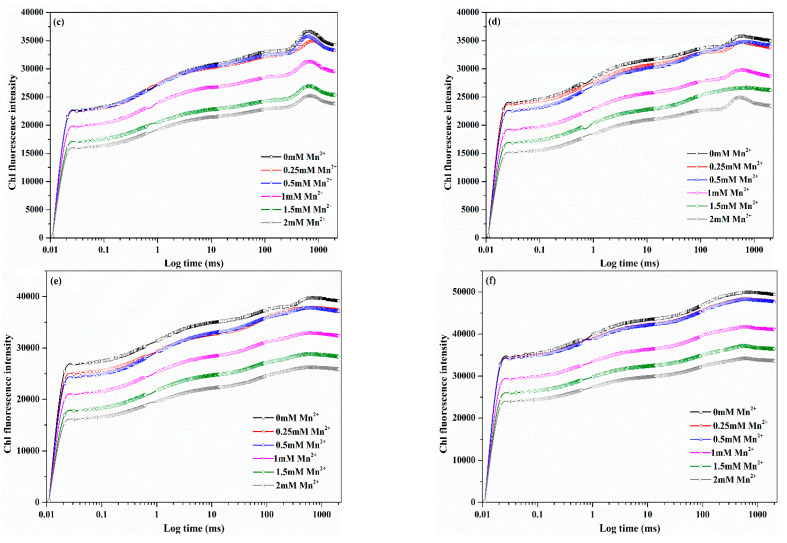
Log scale OJIP chlorophyll fluorescence transients of the mixed MnOMs under different Mn^2+^ concentrations at the cultivation times of (**a**) 0 day, (**b**) 2 days, (**c**) 4 days, (**d**) 6 days, (**e**) 8 days, and (**f**) 10 days.

The variation in OJIP results could represent the photosynthetic activity of microalgae [28]. Thus, the effects of Mn^2+^ on the photosynthetic activity of MnOMs may be revealed by evaluating the OJIP chlorophyll fluorescence transients. As shown in Figure 2, with the Mn^2+^ concentration less than 0.50 mmol/L, the OJIP chlorophyll fluorescence transients of MnOMs at the same cultivation time were similar to the control group. It demonstrated that the Mn^2+^ (0.50 mmol/L) had an insignificant influence on the photosynthetic activity of MnOMs. However, higher Mn^2+^ concentration (0.50–2.00 mmol/L) significantly inhibited the fluorescence yield of MnOMs compared with other treatments. Additionally, the OJIP chlorophyll fluorescence transients under higher Mn^2+^ concentrations varied significantly along the cultivation period. For instance, the fluorescence yields were the lowest, and the shape of the OJIP curves was significantly changed (tended to be a straight line) after 6 days. This finding revealed that the photosynthetic activity, mainly the electron transport chain, was negatively influenced by higher Mn^2+^ concentrations [29]. These results were consistent with the effects of Mn^2+^ on the growth and total chlorophyll content of MnOMs.

#### 3.1.3. Mn^2+^ Oxidation Ability of MnOMs

**Figure 3:**
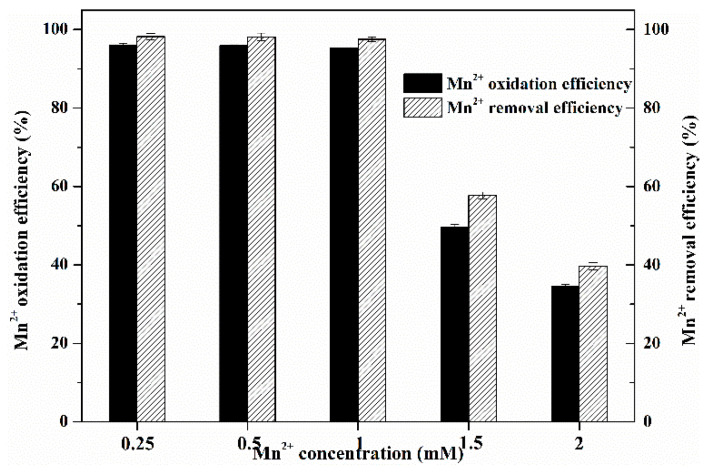
Mn^2+^ oxidation and removal efficiency by mixed MnOMs after 10 days of culturing.

The mixed MnOMs exhibited a high Mn^2+^ oxidation efficiency (>90%) at the Mn^2+^ concentrations of ≤1.00 mmol/L; the Mn^2+^ oxidation efficiency only decreased slightly at the Mn^2+^ concentration range from 0.25 mmol/L to 1.00 mmol/L (Figure 3). However, increasing the Mn^2+^ concentration from 1.00 mmol/L to 2.00 mmol/L significantly decreased the Mn^2+^ oxidation efficiency. We noticed that the Mn^2+^ oxidation efficiency of the mixed MnOMs decreased from 96.12% at the Mn^2+^ concentration of 0.25 mmol/L to 49.78% and 35.56% at Mn^2+^ concentrations of 1.50 mmol/L and 2.00 mmol/L, respectively. In addition, the removal efficiency of Mn^2+^ at each different Mn^2+^ concentration was slightly greater than its oxidation efficiency, indicating that the MnOMs remove Mn^2+^ mainly through the oxidation process rather than by adsorption and uptake or precipitating. The oxidation efficiency of Mn^2+^ decreasing with increasing Mn^2+^ concentration may be caused by the toxic effect of a high Mn^2+^ concentration on MnOMs, which is supported by the effects of Mn^2+^ on MnOMs growth and chlorophyll content.

### 3.2. DCF Removal Efficiency and Pathways

#### 3.2.1. DCF Removal Efficiency

**Figure 4:**
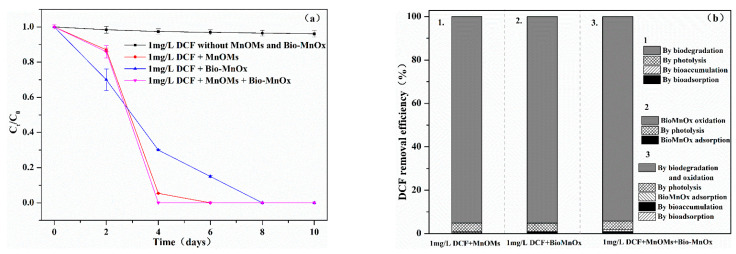
DCF removal efficiency by MnOMs, Bio-MnOx, and a combination of MnOMs and Bio-MnOx; (**a**) variation of the DCF concentration over time; (**b**) DCF removal efficiency by different processes after 10 days.

As depicted in Figure 4, the DCF (1 mg/L) can be completely removed by the mixed MnOMs and Bio-MnOx within 10 days, demonstrating the abilities of both MnOMs and Bio-MnOx for DCF removal. It is worth noting that the combination of MnOMs and Bio-MnOx could significantly (*p <* 0.05) increase DCF (1 mg/L) removal rate from 0.167 ± 0.008 mg/L·d (by MnOMs alone) and 0.125 ± 0.024 mg/L·d (by Bio-MnOx alone) to 0.250 ± 0.016 mg/L·d. The removal rate of DCF by Bio-MnOx was higher in the initial 2 days than in MnOMs or the MnOMs/Bio-MnOx, mainly due to the requirement of time for MnOMs growth and the production of Bio-MnOx in these two reactions. DCF removal efficiency in the control group without MnOMs and Bio-MnOx did not exceed 4%, which indicated that the removal of DCF by the photo-degradation process was negligible. Microalgae can remove PhCs by bioadsorption, bioaccumulation, and biodegradation [17], whereas manganese oxides remove PhCs mainly through adsorption and oxidative degradation [5]. The quantity of DCF absorbed and accumulated within the MnOMs cells was less than 1%, and the removal efficiency of DCF by adsorption of Bio-MnOx also did not exceed 1% (Figure 4b). These results indicated that the MnOMs and Bio-MnOx remove DCF mainly through biodegradation and oxidation degradation, respectively.

#### 3.2.2. DCF Removal Pathway

**Figure 5:**
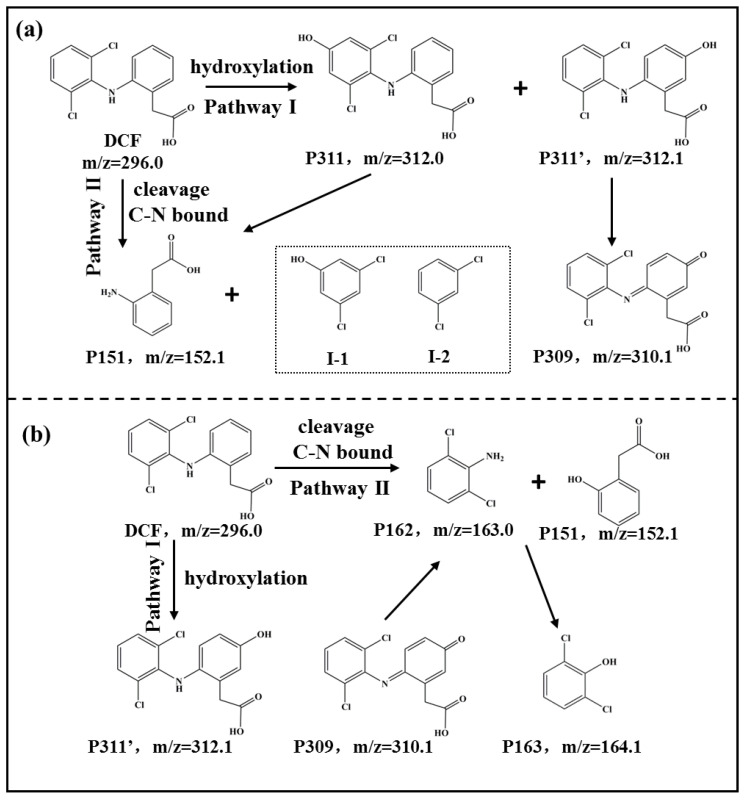
Proposed pathways of DCF degradation by (**a**) MnOMs and (**b**) Bio-MnOx.

Five, four, and three types of intermediates were obtained during the DCF degradation process by MnOMs, Bio-MnOx, and MnOMs/Bio-MnOx, respectively (Table 1). Plausible DCF degradation pathways by MnOMs and Bio-MnOx were proposed based on product identification, as depicted in Figure 5. The MnOMs degraded DCF via two main pathways (Figure 5a). In pathway I, DCF was hydroxylated first to form P311 and P311’. The DCF hydroxylation products P311 and P311’ are the main products of DCF degradation by many bacteria, fungi, and microalgae and the main metabolites of DCF in the human body [2,30,31]. For example, Ouada et al. [2] suggested that the microalgae *Picocystis* sp. degrades DCF to P311. P311 is also the main degradation product of DCF by *Bacillus subtilis*, *Actinoplanes* sp., *Raoultella* sp., KDF8, and *L. Portucalensis*; these bacteria hydroxylate DCF mainly through a series of different enzymes in CYP450 [30]. In addition, Pylypchuk et al. [31] showed that laccase and lignin peroxidase can hydroxylate DCF to form P311 and P311’. Therefore, the MnOMs may also metabolize DCF into P311 and P311’ by producing CYP450 or other types of peroxidase. The C-N bond in P311 can be broken to form P151 and I-1 under the action of enzymes, whereas P311’ can be further oxidized to form P309 under the action of peroxidase or other enzymes. On the other hand, DCF directly generates P151 and I-2 by the C-N bond cleavage in pathway II.

The pathway of DCF degradation by as-prepared Bio-MnOx was similar to some synthetic chemical MnO_2_ and other Bio-MnOx produced by manganese-oxidizing bacteria [23,32]. As shown in Figure 5b, DCF can form P311’ through hydroxylation in pathway I. The formed P311’ can be further oxidized to form P309, which can further form P162 after the cleavage of the C-N bond. P162 can then undergo hydroxylation to form P163. In pathway II, DCF can also directly form P162 and P151 through the cleavage of the C-N bond, and then the formed P162 undergoes further hydroxylation to form P163.

### 3.3. Synergistic Effects of MnOMs and Bio-MnOx

**Figure 6:**
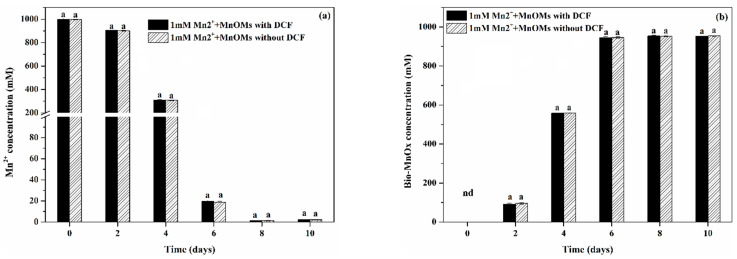
Concentration variation of (**a**) Mn^2+^ and (**b**) Bio-MnOx over time. The same letters above the error bars showed insignificant differences between control and treatment (*p* < 0.05).

Although either the MnOMs or Bio-MnOx working alone can completely degrade 1 mg/L of DCF within 8 days, the combination of MnOMs and Bio-MnOx exhibits a much higher DCF removal rate. This result indicates that there may be some synergistic effects of MnOMs and Bio-MnOx for DCF removal. Tran et al. [15] reported that the MOB *Pseudomonas putida* strain MnB1 could enhance Bio-MnOx degrade 17α-ethinylestradiol through re-oxidizing Mn(II) to Bio-MnOx. Wang et al. [21] also indicated that the green algae *Desmodesmus* sp. WR1 can improve bisphenol A removal by Bio-MnOx using a similar mechanism. In our present study, the concentration of Mn^2+^ and Bio-MnOx at each cultivation time in the DCF removal process combining MnOMs and Bio-MnOx was insignificant (*p* *<* 0.05) in the reaction system without DCF (Figure 6). This finding suggested that the Mn^2+^ produced from the redox of Bio-MnOx and DCF could be re-oxidized to Bio-MnOx again by MnOMs. This process can continuously provide Bio-MnOx as the oxidant and reduce the occupation of Mn^2+^ at the reaction site. The regeneration of Bio-MnOx driven by MnOMs could effectively facilitate the removal of DCF. The synergistic effect of Bio-MnOx and MnOMs could be one of the origins of the superior performance of the MnOMs/Bio-MnOx process.

The DCF degradation intermediates generated in the DCF degradation by MnOMs, Bio-MnOx, and MnOMs/Bio-MnOx were compared, and it is worth noting that some degradation intermediates detected in the DCF degradation by MnOMs or Bio-MnOx were not observed in the DCF degradation by the MnOMs/Bio-MnOx (Table 1). For example, P311 and P150 generated in the DCF degradation process by MnOMs were not detected in the DCF removal by MnOMs/Bio-MnOx. P311' and P162 generated in the DCF degradation process by Bio-MnOx were also not detected in the process with the presence of MnOMs/Bio-MnOx. These results indicate that the DCF degradation intermediates by MnOMs and Bio-MnOx may be exchanged as mutual reactants to be further degraded, meaning that the intermediates, which Bio-MnOx can hardly oxidize, could be degraded by MnOMs. By contrast, the pathway of DCF degradation by MnOMs is also optimized with the presence of Bio-MnOx. Thus, this synergistic effect of MnOMs and Bio-MnOx could effectively contribute to the DCF removal, resulting in improved performance.

## 4. Conclusions

The mixed MnOMs had a high Mn^2+^ tolerance and oxidation efficiency (>85%) for Mn^2+^ concentrations ranging from 0.25 to 1.00 mmol/L. This low Mn^2+^ concentration did not influence the growth, chlorophyll content, or photosynthetic activity of MnOMs. However, increasing the Mn^2+^ concentration (1.00–2.00 mmol/L) seriously inhibited the growth of MnOMs. The combination of MnOMs and Bio-MnOx significantly improved the DCF (1 mg/L) removal rate from 0.167 ± 0.008 to 0.125 ± 0.024 mg/L·d (for MnOMs and Bio-MnOx alone, respectively) to 0.250 ± 0.016 mg/L·d. The synergistic effects of MnOMs and Bio-MnOx in the DCF degradation process may be (i) the Mn^2+^ re-oxidation by MnOMs, and (ii) the mutual exchange of DCF degradation products as shared reactants between MnOMs and Bio-MnOx. The DCF removal by MnOMs/Bio-MnOx is attributed to biodegradation and chemical oxidation degradation. The DCF degradation pathway by MnOMs and Bio-MnOx was proposed based on the identification of intermediates.

## Figures and Tables

**Table 1:** Tentative identification of DCF degradation metabolites by microalgae, BioMnOx, and the combined action.

Degradation Products				Reaction Systems		
Name	Retention Time (min)	Structural Formula	*m*/*z* [M + H]^+^	MnOMs + DCF	BioMnOx + DCF	MnOMs + BioMnOx + DCF
DCF	5.212	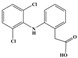	296.0	√	√	√
P311	4.322	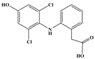	312.0	√	nd	nd
P311’	2.886	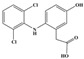	312.1	√	√	nd
P150	9.547	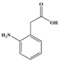	151.1	√	nd	nd
P309	10.175	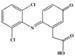	310.1	√	√	√
P151	8.754	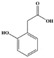	152.1	nd	√	√
P162	11.634	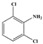	163.0	nd	√	nd
P163	12.052	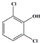	164.1	nd	√	√

Note: “√” means detected in the reaction system and “nd” means not detected.

## Data Availability

The data presented in this article are available on request from the corresponding authors.

## Supplementary Materials

The following supporting information can be downloaded at: https://www.mdpi.com/article/10.3390/toxics10050230/s1, Text S1: The composition and content of the BG11 medium; Text S2: Sep-aration and preparation of Bio-MnOx; Text S3: Measurement of total chlorophyll and photosyn-thetic activity; Text S4: Measurement of DCF and product Characterization; Text S5: Method of distinguishing bioabsorption, bioaccumulation, and biodegradation of DCF by MnOMs; Figure S1: The XRD pattern of Bio-MnOx generated by the mixed MnOMs; Figure S2: FE-SEM images of (a) the mixed MnOMs, and (b) Bio-MnOx generated by the mixed MnOMs. References [17,33,34] are cited in the supplementary materials.
