# Supplementary material for: Combined Process of Biogenic Manganese Oxide and Manganese-Oxidizing Microalgae for Improved Diclofenac Removal Performance: Two Different Kinds of Synergistic Effects
Source: Toxics. 2022 Apr 30;10(5):230. doi: 10.3390/toxics10050230 (PMC9147876; doi:10.3390/toxics10050230)
Supplement: Supplementary file 1 [file toxics-10-00230-s001.zip › toxics-1676505-supplementary.pdf]

toxics MDPI

Supplementary Materials: Combined Process of Biogenic Manganese Oxide and Manganese-Oxidizing Microalgae for Improved Diclofenac Removal Performance: Two Different Kinds of Synergistic Effects

Quanfeng Wang, Cenhui Liao, Jujiao Zhao, Guoming Zeng, Wenbo Liu, Pei Gao, Da Sun and Juan Du

Text S1: The composition and content of the BG-11 medium

The BG-11 medium consists of 1.500 g $NaNO_3$, 0.040 g K_2HPO_4, 0.075 g $MgSO_4.7H_2O$, 0.036 g $CaCl_2{\cdot}2H_2O$, 0.006 g citric acid, 0.006 g ammonium ferric citrate, 0.001 g Na_2-EDTA, 0.200 g Na_2CO_3, and 1 mL A5 trace metal solution per liter. An A5 trace metal solution was prepared using deionized water as a solvent, and 1 L of A5 trace metal solution contains 2.860 g H_3BO_3, 1.810 g $MnCl_2{\cdot}4H_2O$, 0.222 g $ZnSO_4{\cdot}7H_2O$, 0.390 g Na_2MoO_4, 0.079 g $CuSO_4{\cdot}5H_2O$, and 0.049 g $Co(NO_3)_2{\cdot}6H_2O$. The culture medium was autoclaved, and the pH was adjusted to 7.0 using 0.1 M HCl and 0.1 M NaOH.

Text S2: Separation and preparation of Bio-MnOx

To investigate DCF removal by the single Bio-MnOx, the mixed MnOMs were first grown in 100 mL of BG-11 medium with 1 mM Mn^{2+}. After 10 d of incubation, the MnOMs were segregated with Bio-MnOx by a cell lysis method, which is similar to the method of total chlorophyll measurement [1]. In brief, the microalgal suspension was sampled and centrifuged at 15000 rpm for 15 min. After discarding the supernatant, the mixture of microalgal pellets and Bio-MnOx was performed in four cycles of freezing and thawing (a cycle is frozen at -20°C for 4 hours and thawed at room temperature for 20 min) to disrupt the cell wall. A total of 20 mL of ethanol was then added to the mixture, and the mixture was kept in an 80°C water bath for 15 min. Afterward, the Bio-MnOx was separated from the suspension by centrifugation at 15000 rpm for 15 min and washed twice with ethanol and deionized water, respectively. The generated Bio-MnOx was harvested by centrifugation and freeze-drying for the subsequent experiments.

Text S3: Measurement of total chlorophyll and photosynthetic activity

The total chlorophyll and carotenoid contents in the S. platensis were extracted by 2 mL of 96% ethanol and measured at 665, 649, and 470 nm. The chlorophyll and carotenoids concentration can be calculated by the equation described by Danesh et al. [2]. Briefly, 1 mL (V_1) of microalgal suspension was sampled and centrifuged at 15000 rpm for 15 min. After discarding the supernatant, the microalgal pellets underwent four cycles of freezing and thawing (a cycle was frozen at -20°C for 4 hours and thawed at room temperature for 20 min) to disrupt the cell wall. A certain volume of ethanol (V_2) was then added to the pellet, mixed well, and kept in a boiling water bath for 15 min. After centrifuging at 15000 rpm for 15 min, the absorbance of the supernatant was measured at 665, 649, and 470 nm using a DR 2800 UV-Vis spectrophotometer (Hach, USA). The chlorophyll and carotenoid concentration in the sample could be obtained by the Equations (S1)–(S3) [2]:

$$C_a(mg/L) = \frac{(13.95\,A_{665} - 6.88\,A_{649}) \times V_2}{V_1} \quad (S1)$$

$$C_b(mg/L) = \frac{(24.96\,A_{649} - 7.32\,A_{665}) \times V_2}{V_1} \quad (S2)$$

$$C_t(mg/L) = C_a + C_b \quad (S3)$$

where V_1 and V_2 are volumes of sampled microalgae suspension and latter supernatant, respectively. A_{665} and A_{649} are the absorbances of the supernatant at 665 and 649 nm, respectively. C_a, C_b, and C_t indicate the concentration of chlorophyll a, chlorophyll b, and total chlorophyll, respectively.

In order to test the photosynthetic activity of MnOMs, the microalgae suspension was adjusted to a certain optical density (0.2±0.05) with distilled water before testing. After adapting to darkness for 20 min, the OJIP test of the microalgal cells was measured by the portable fluorometer (AquaPen-C 100, Photon Systems Instruments, Czech Republic), and FluorPen 1.0 software was used for data analysis. The Q_A model and energy fluxes reported by Strasser et al. were used for Chl fluorescence analysis in this study [3].

Text S4: Measurement of DCF and product Characterization

We analyzed the DCF concentrations using an Agilent 1260 HPLC system (Agilent Technologies, USA) coupled with a Zorbax Eclipse XDB-C18 column (2.1×150 mm, 3.5 μm, Agilent) and a diode array UV-Vis detector. The column temperature was maintained at 25°C during the sample analysis. The isocratic mobile phase consisted of acetonitrile and 0.5% acetic acid solution (45:55, V/V) at a 0.8 mL/min flow rate. The detection wavelength was set at 276 nm, and the injection volume was 20 μL. The retention time for DCF in the HPLC system was 5.5 min, and the detection limit was 0.02 mg/L.

For the characterization of DCF degradation by HPLC/MS/MS, the Agilent G1316B Column-SL (2.1 × 50 mm, 1.9 μm) was used for HPLC. The eluent consisted of two mobile phases at 0.2 mL/min: (A) 0.1% formic acid in water (v/v) and (B) 100% acetonitrile. The gradient was as follows: component A was maintained at 95% during the first 2 min, then B linearly increased from 5 to 85% in the next 10 min, and B was maintained at 85% in the next 10 min. Mass spectrometric analysis was conducted using positive electrospray ionization with a mass scan range of m/z 50–1000. The spray voltage, vaporizer temperature, sheath gas pressure, aux gas pressure, and capillary temperature were set at 4000 V, 350°C, 40 psig, 10 psig, and 320°C, respectively.

Text S5: Method of distinguishing bioabsorption, bioaccumulation, and biodegradation of DCF by MnOMs

The DCF quantification of residual in the medium, adsorbed on the surface of microalgae cells, and bioaccumulated in microalgal cells, could be obtained according to the method reported in our previous study [1]. In brief, the microalgal suspensions were directly syringe filtered through 0.22 μm nylon filters to detect the residual DCF in the medium. In order to measure the DCF adsorbed on the microalgal cells' surface, the microalgal cell pellets were harvested by centrifugation at 15000 rpm. Subsequently, the microalgal cell pellets were washed three times using 10 mL of ethanol–water (1:9 v/v), collected after centrifugation as the supernatant, and used to analyze DCF adsorbed on the surfaces of microalgal cells. After washing with ethanol–water, the pellets were harvested and mixed with 3 mL of anhydrous ethanol. After sonication for 1 h, the sample was centrifuged for 10 min at 15000 rpm, and the supernatant was used for DCF quantification accumulated within the microalgal cells.

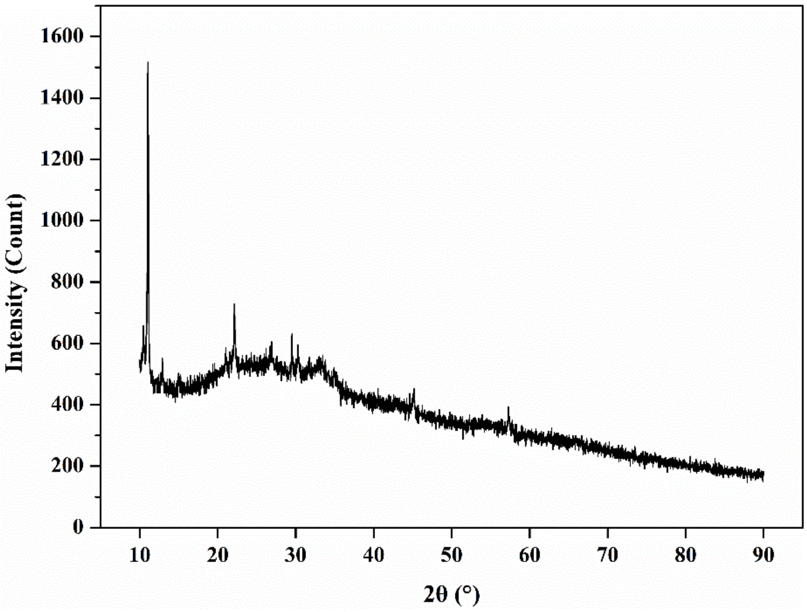

Figure S1. The XRD pattern of Bio-MnOx generated by the mixed MnOMs.

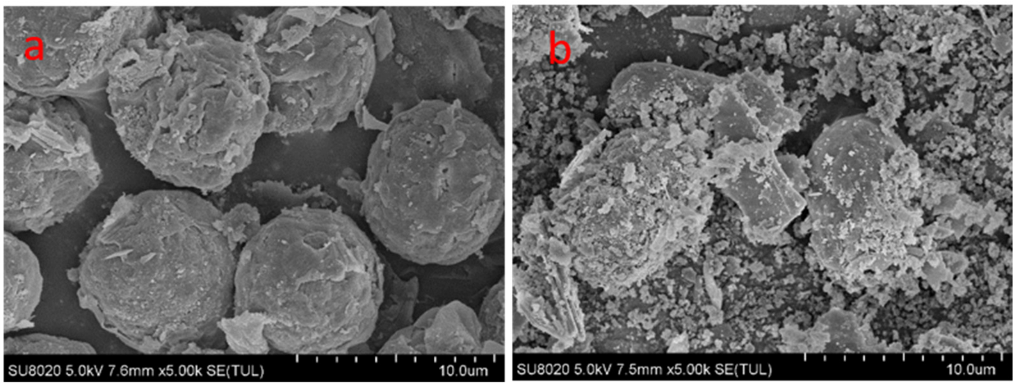

Figure S2. FE-SEM images of (**a**) the mixed MnOMs, and (**b**) Bio-MnOx generated by the mixed MnOMs.

References

1. Wang, Q.; Liu, W.; Li, X.; Wang, R.; Zhai, J. Carbamazepine toxicity and its co-metabolic removal by the cyanobacteria Spirulina platensis. *Sci. Total Environ.* **2020**, *706*, 135686.
2. Danesh, A.F.; Ebrahimi, S.; Salehi, A.; Parsa, A. Impact of nutrient starvation on intracellular biochemicals and calorific value of mixed microalgae. *Biochem. Eng. J.* **2017**, *125*, 56–64.
3. Strasser, R.J.; Tsimilli-Michael, M.; Dangre, D.; Rai, M. Biophysical phenomics reveals functional building blocks of plants systems biology: A case study for the evaluation of the impact of mycorrhization with Piriformospora indica. In *Soil Biology*; Varma, A., Oelmuller, R., Eds.; Springer: Berlin/Heidelberg, Germany, 2007; Volume 11, pp. 319–341.
